# Necroptosis increases with age and is reduced by dietary restriction

**DOI:** 10.1111/acel.12770

**Published:** 2018-04-25

**Authors:** Sathyaseelan S. Deepa, Archana Unnikrishnan, Stephanie Matyi, Niran Hadad, Arlan Richardson

**Affiliations:** ^1^ Department of Geriatric Medicine Oklahoma University Health Science Center Oklahoma City OK USA; ^2^ The Reynolds Oklahoma Center on Aging Oklahoma City OK USA; ^3^ Oklahoma Center for Neuroscience Oklahoma University Health Science Center Oklahoma City OK USA; ^4^ Oklahoma City VA Medical Center Oklahoma City OK USA

**Keywords:** adipose tissue, aging, dietary restriction, inflammaging, inflammation, necroptosis

## Abstract

Necroptosis is a newly identified programmed cell death pathway that is highly proinflammatory due to the release of cellular components that promote inflammation. To determine whether necroptosis might play a role in inflammaging, we studied the effect of age and dietary restriction (DR) on necroptosis in the epididymal white adipose tissue (eWAT), a major source of proinflammatory cytokines. Phosphorylated MLKL and RIPK3, markers of necroptosis, were increased 2.7‐ and 1.9‐fold, respectively, in eWAT of old mice compared to adult mice, and DR reduced P‐MLKL and P‐RIPK3 to levels similar to adult mice. An increase in the expression of RIPK1 (1.6‐fold) and MLKL (2.7‐fold), not RIPK3, was also observed in eWAT of old mice, which was reduced by DR in old mice. The increase in necroptosis was paralleled by an increase in 14 inflammatory cytokines, including the pro‐inflammatory cytokines IL‐6 (3.9‐fold), TNF‐α (4.7‐fold), and IL‐1β (5.1‐fold)], and 11 chemokines in old mice. DR attenuated the expression of IL‐6, TNF‐α, and IL‐1β as well as 85% of the other cytokines/chemokines induced with age. In contrast, inguinal WAT (iWAT), which is less inflammatory, did not show any significant increase with age in the levels of P‐MLKL and MLKL or inflammatory cytokines/chemokines. Because the changes in biomarkers of necroptosis in eWAT with age and DR paralleled the changes in the expression of pro‐inflammatory cytokines, our data support the possibility that necroptosis might play a role in increased chronic inflammation observed with age.

Aging is characterized by the progressive increase in chronic, low‐grade inflammation termed “inflammaging,” which is believed to play an important role in the mechanism underlying aging (Franceschi & Campisi, 2014). Necroptosis is a newly identified form of cell death that initiates an inflammatory process when the dying cells release cell debris and self‐molecules, that is, damage‐associated molecular patterns, DAMPs or alarmins (Pasparakis & Vandenabeele, 2015). DAMPs are a major activator of NLRP3 inflammasome that triggers maturation of interleukin‐1β (IL‐1β), and NLRP3 inflammasome activation is one of the mechanisms that induces low‐grade chronic inflammation with age (Camell et al., 2017; Furman et al., 2017). Necroptosis is triggered when stimuli induce the phosphorylation of receptor interacting serine/threonine kinase 1 (RIPK1), leading to the phosphorylation of RIPK3 and MLKL. Phosphorylated MLKL (P‐MLKL) oligomerizes, binds to, and ruptures the cell membrane, resulting in the release of cellular components including DAMPs. Several studies show that blocking necroptosis either genetically or pharmacologically dramatically reduces inflammation in a variety of mouse models (Ito et al., 2016; Meng et al., 2015). In addition, blocking/reducing necroptosis appears to have an impact on the aging of the male reproductive system (Li et al., 2017) and increases the lifespan of *ApoE* knockout mice (Meng et al., 2015) and G93A transgenic mouse model of ALS (Ito et al., 2016). Necroptosis also appears to play a role in neuron loss in Alzheimer's disease (Caccamo et al., 2017).

To determine whether necroptosis might be a factor in inflammaging, we determined whether necroptosis increases with age and whether it was attenuated by DR, which retards aging and reduces the increase in chronic inflammation (Spaulding et al., 1997). We measured necroptosis in eWAT, which is a visceral fat depot that is associated with the greatest inflammatory cytokine production, compared to other fat depots, and iWAT, which is a subcutaneous fat depot less inflammatory in nature (Huffman & Barzilai, 2009). As shown in Figure 1a, the level of P‐MLKL, a well‐accepted marker of necroptosis, was 2.7‐fold greater in eWAT of old mice (25–29 months) compared to adult mice (9 months), and DR (started at 4 months of age) reduced P‐MLKL to a level similar to adult mice. MLKL protein and transcript levels were also higher in eWAT of old mice (2.7‐ and 2.2‐fold, respectively), whereas MLKL levels in old mice on DR were similar to adult mice (Figures 1a, b). Phosphorylation of RIPK3, the upstream kinase of MLKL, was elevated in old mice (1.9‐fold) and DR reduced RIPK3 phosphorylation in old mice. However, protein expression of RIPK3 was comparable in adult, old and old mice fed DR diet (Figure 1a). Similarly, protein expression of RIPK1, upstream kinase of RIPK3, was elevated 1.6‐fold in old mice, compared to adult mice and DR reduced its expression (Figure 1a). The transcript levels of RIPK3 were similar in adult, old and old‐DR mice; however, the level of RIPK1 transcript was increased in eWAT of old mice (2.3‐fold) (Figure 1b). A similar increase in the transcript levels of RIPK1 and MLKL was reported in the brain of patients with AD (Caccamo et al., 2017). Under conditions of necroptotic cell death, ESCRT‐III controls the duration of plasma membrane integrity (Gong et al., 2017). We found no significant change in the transcript levels of ESCRT components (VPS4A, VPS4B, VPS37B, CHMP4B, and CHMP2A) in adult and old eWAT (Figure 1c), suggesting that cells in old mice are not protected from necroptotic cell death by this pathway. In contrast, iWAT did not show any significant increase with age in the protein levels of P‐MLKL, MLKL, P‐RIPK3, RIPK3, and RIPK1 or the transcript levels of RIPK1, RIPK3, or MLKL (Figures 1d–f).

**Figure 1:**
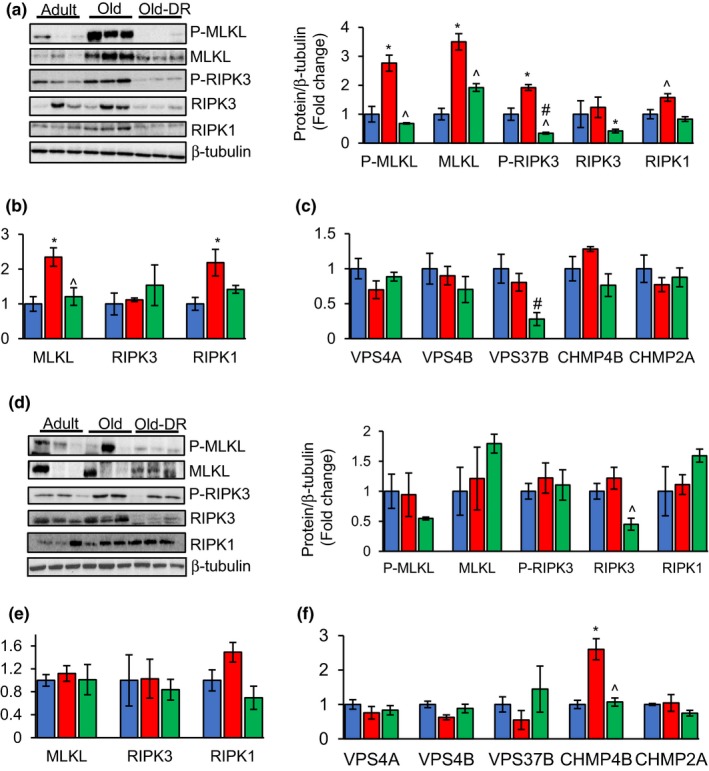
Markers of necroptosis increase with age in eWAT, not iWAT, and DR reduces their expression in eWAT. (a, d) Left panel: immunoblots of eWAT (a) and iWAT (d) extracts from adult (blue bar), old (red bar), and old‐DR (green bar) mice for P‐MLKL, MLKL, P‐RIPK3, RIPK3, RIPK1, and β‐tubulin (*n* = 5–6/group). Right panel: graphical representation of quantified blots normalized to β‐tubulin. (b, e) Transcript levels of MLKL, RIPK3, and RIPK1 in eWAT (b) and iWAT (e) of adult, old, and old‐DR mice, normalized to β‐microglobulin and represented as fold change. (c, f) Transcript levels of VPS4A, VPS4B, VPS37B, CHMP4B, and CHMP2A in eWAT (c) and iWAT (f) of adult, old, and old‐DR mice, normalized to β‐microglobulin and represented as fold change. Data shown are mean ± *SEM*. *p* < .05 is taken as significant for the following: *Adult vs old; ^old vs old‐DR; #adult vs old‐DR

We next determined whether the increase in necroptosis in eWAT was associated with increased inflammation. DAMPs produced by necroptosis are reported to increase the release of pro‐inflammatory cytokines from innate immune cells through the activation of NF‐κB (Land, 2015). Therefore, we measured activation of NF‐κB in eWAT by the phosphorylation of NF‐κB p65 (pS536). As shown in Figure 2a, the level of phospho‐NF‐κB normalized to NF‐κB was 1.4‐fold greater in eWAT of old mice compared to adult mice, and DR reduced phospho‐NF‐κB to a level similar to adult mice. We next measured the transcript levels of 61 cytokines and chemokines in eWAT and iWAT from adult, old, and old‐DR mice (Figure 2b left panel and Table S1). Of the 35 inflammatory cytokines analyzed, 14 showed significant increase with age and 11 of the 26 chemokines analyzed showed an increase with age in eWAT. DR significantly attenuated the expression 21 of the 25 cytokines/chemokines that increased with age (Figure 2b, left panel and Table S1). In iWAT, seven of the 61 cytokines/chemokines showed a significant change in their expression with age and DR altered the expression of only three cytokines/chemokines (Figure 2b, right panel and Table S1). The data in Figure 2c show that the transcript levels of the pro‐inflammatory cytokines IL‐6, TNF‐α, and IL‐1β, which are strong predictors of mortality and chronic diseases in elderly humans (Singh & Newman, 2011) and have been shown to be induced by necroptosis (Ito et al., 2016), are significantly increased in eWAT from old mice 3.9‐, 4.7‐, and 5.1‐fold, respectively, and are attenuated by DR.

**Figure 2:**
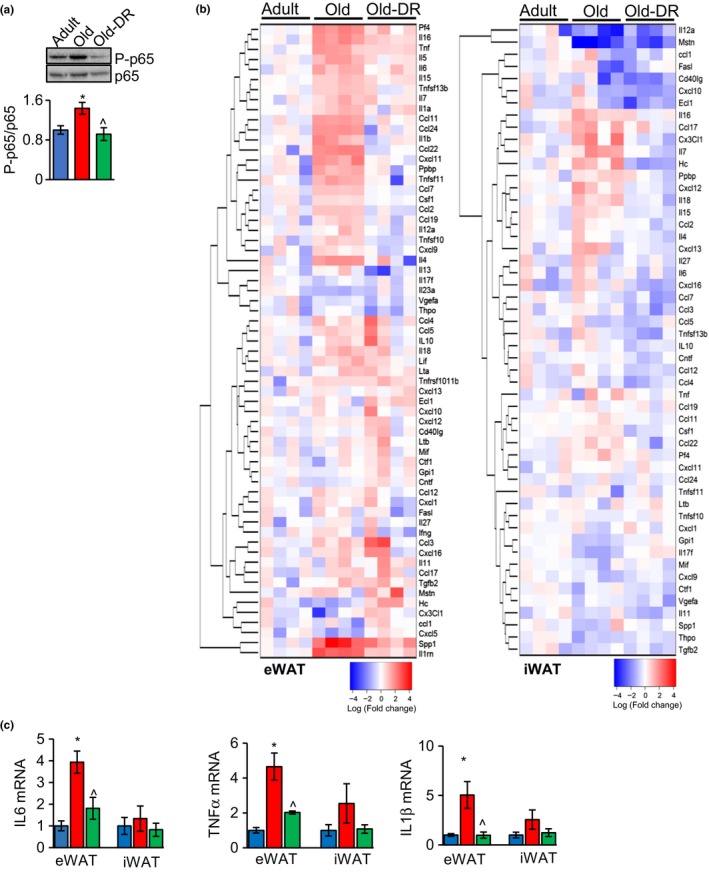
Transcript level of inflammatory markers increases with age in eWAT, not in iWAT, and DR reduces the expression of inflammatory markers in eWAT of old mice. (a) Top panel: immunoblots of eWAT extracts from adult (blue bar), old (red bar), and old‐DR (green bar) mice for phospho‐NF‐κB and NF‐κB (*n* = 5–6/group). Bottom panel: graphical representation of quantified blots of phospho‐NF‐κB normalized to NF‐κB. (b) Heat maps showing expression of inflammatory cytokines and chemokines, normalized to β‐microglobulin in eWAT (left panel) and iWAT (right panel) of adult, old, and old‐DR mice (*n* = 4/group). Average value of adult mice was used to normalize values of adult, old, and old‐DR mice. The darker the red indicates the greater the increase in expression, and the darker the blue indicates the greater the decrease in expression. (c) Transcript levels of IL‐6, TNF‐α, and IL‐1β in eWAT and iWAT of adult (blue bar), old (red bar), and old‐DR (green bar) mice. Transcript levels in adult eWAT and iWAT were taken as one, and fold changes relative to adult are represented. Data shown are mean±SEM. The data were analyzed by one‐way ANOVA, and a *p* < .05 is taken as significant for the following: *adult vs old; ^old vs old‐DR

In summary, our study is the first to demonstrate that biomarkers of necroptosis increase with age. The observation that the changes in necroptosis in eWAT with age and DR are paralleled by changes in the expression of pro‐inflammatory cytokines support the possibility that necroptosis may play a role in the age‐related increase in chronic inflammation in visceral fat, and possibly inflammaging in the whole animal. Using genetic and pharmacological manipulations, which block necroptosis, it will be possible to determine whether the age‐related increase in necroptosis causes the increased inflammation observed with age.

## CONFLICT OF INTEREST

None declared.
