# Supplementary material for: Necroptosis increases with age and is reduced by dietary restriction
Source: Aging Cell. 2018 Apr 25;17(4):e12770. doi: 10.1111/acel.12770 (PMC6052392; doi:10.1111/acel.12770)
Supplement: Supplementary file 1 [file ACEL-17-na-s001.pdf]

Supplementary Table I. Changes in cytokine/chemokine gene expression in adult, old and old-DR eWAT and iWAT

eWAT										
		**Young**		**Old**		**Old-DR**		**Significance**	**Significance**	**Significance**
**Gene**	**Gene Name**	**Fold change**	**SEM**	**Fold change vs young**	**SEM**	**Fold change vs young**	**SEM**	**Young vs Old**	**Young vs Old-DR**	**Old vs Old-DR**
Pf4	Platelet Factor 4	1	0.12	6.95	0.66	2.35	0.66	Yes	No	Yes
Il16	Interleukin 16	1	0.19	6.01	0.78	2.11	0.22	Yes	Yes	Yes
Tnf	Tumor Necrosis Factor	1	0.16	4.66	0.78	2.02	0.09	Yes	Yes	Yes
Il5	Interleukin 5	1	0.19	4.40	0.64	1.56	0.28	Yes	No	Yes
Il6	Interleukin 6	1	0.23	3.93	0.51	1.81	0.50	No	No	No
Il15	Interleukin 15	1	0.08	2.62	0.24	1.69	0.50	Yes	Yes	Yes
Tnfsf13b	TNF Superfamily Member 13b	1	0.12	2.54	0.19	1.26	0.41	Yes	No	Yes
Il7	Interleukin 7	1	0.27	2.84	0.64	1.68	0.47	Yes	No	No
Il1a	Interleukin 1alpha	1	0.12	2.86	0.43	1.54	0.46	Yes	No	No
Ccl11	C-C Motif Chemokine Ligand 11	1	0.32	5.11	0.28	1.30	0.37	Yes	Yes	Yes
Ccl24	C-C Motif Chemokine Ligand 24	1	0.25	5.96	0.22	0.88	0.34	Yes	No	Yes
Il1b	Interleukin 1beta	1	0.14	5.05	1.35	0.99	0.31	Yes	No	Yes
Ccl22	C-C Motif Chemokine Ligand 22	1	0.30	7.78	0.40	0.76	0.15	Yes	No	Yes
Cxcl11	C-X-C Motif Chemokine Ligand 11	1	0.35	6.23	1.17	1.09	0.22	Yes	No	Yes
Ppbp	Pro-Platelet Basic Protein	1	0.37	4.01	0.42	1.00	0.32	Yes	No	Yes
Tnfsf11	TNF Superfamily Member 11	1	0.35	4.18	0.47	0.96	0.31	Yes	No	Yes
Ccl7	C-C Motif Chemokine Ligand 7	1	0.22	2.69	0.10	0.80	0.09	Yes	No	Yes
Csf1	Colony Stimulating Factor 1	1	0.30	1.80	0.03	0.90	0.05	Yes	No	Yes
Ccl2	C-C Motif Chemokine Ligand 2	1	0.30	2.75	0.06	0.72	0.22	Yes	No	Yes

Ccl19	C-C Motif Chemokine Ligand 19	1	0.34	2.16	0.23	0.92	0.28	Yes	No	Yes
Il12a	Interleukin 12a	1	0.19	1.97	0.59	0.86	0.22	No	No	No
Tnfsf10	TNF Superfamily Member 10	1	0.43	2.23	0.25	0.56	0.06	Yes	No	Yes
Cxcl9	C-X-C Motif Chemokine Ligand 9	1	0.48	1.71	0.23	0.78	0.21	No	No	Yes
Il4	Interleukin 4	1	0.43	6.18	0.40	1.56	1.05	Yes	No	Yes
Il13	Interleukin 13	1	0.34	1.55	0.49	0.34	0.17	No	No	No
Il17f	Interleukin17f	1	0.25	0.67	0.13	0.89	0.29	No	No	No
Il23a	Interleukin 23a	1	0.23	0.31	0.03	0.86	0.29	Yes	No	No
Vgefa	Vascular Endothelial Growth Factor A	1	0.48	0.65	0.06	0.55	0.14	No	No	No
Thpo	Thrombopoietin	1	0.54	0.84	0.12	0.38	0.14	No	No	Yes
Ccl4	C-C Motif Chemokine Ligand4	1	0.18	2.13	0.56	4.55	2.87	No	Yes	No
Ccl5	C-C Motif Chemokine Ligand 5	1	0.20	2.14	0.26	3.05	1.89	Yes	No	No
IL10	Interleukin10	1	0.33	1.84	0.38	4.17	2.91	No	No	No
Il18	Interleukin 18	1	0.25	2.82	0.66	2.00	0.66	No	No	No
Lif	LIF, Interleukin 6 Family Cytokine	1	0.23	2.83	0.57	1.59	0.35	Yes	No	No
Lta	Lymphotoxin Alpha	1	0.34	2.07	0.59	2.00	0.47	No	No	No
Tnfrsf11b	TNF Receptor Superfamily Member 11b	1	0.29	1.89	0.03	2.33	0.49	Yes	No	No
Cxcl13	C-X-C Motif Chemokine Ligand 13	1	0.43	1.14	0.19	1.66	0.36	No	No	No
Xcl1	X-C Motif Chemokine Ligand 1	1	0.15	1.25	0.41	2.56	0.45	No	No	No
Cxcl10	C-X-C Motif Chemokine Ligand 10	1	0.25	1.08	0.31	2.84	1.74	No	Yes	No
Cxcl12	C-X-C Motif Chemokine Ligand 12	1	0.22	1.36	0.05	1.78	0.59	No	No	No
Cd40Ig	CD40 Ligand	1	0.28	0.96	0.12	1.94	0.70	No	No	No
Ltb	Lymphotoxin Beta	1	0.32	1.37	0.31	2.25	1.09	No	No	No

Mif	Macrophage Migration Inhibitory Factor	1	0.31	1.18	0.35	1.77	0.72	No	No	No
Ctf1	Cardiotrophin 1	1	0.19	0.95	0.34	1.79	0.44	No	No	No
Gpi1	Glucose-6-Phosphate Isomerase 1	1	0.17	0.90	0.11	1.75	0.46	No	No	No
Cntf	Ciliary Neurotrophic Factor	1	0.32	0.65	0.08	1.48	0.28	No	No	Yes
Ccl12	C-C Motif Chemokine Ligand 12	1	0.23	1.73	0.35	1.32	0.52	No	No	No
Cxcl1	C-X-C Motif Chemokine Ligand 1	1	0.30	1.56	0.24	1.35	0.80	No	No	No
Fasl	Fas Ligand	1	0.37	1.45	0.46	0.83	0.32	No	No	No
Il27	Interleukin 27	1	0.38	1.00	0.22	1.33	0.53	No	No	No
Ifng	Interferon Gamma	1	0.31	1.31	0.37	1.29	0.94	No	No	No
Ccl3	C-C Motif Chemokine Ligand 3	1	0.24	2.39	0.48	9.54	5.27	Yes	No	No
Cxcl16	C-X-C Motif Chemokine Ligand 16	1	0.38	1.91	0.35	3.89	1.95	No	No	No
Il11	Interleukin 11	1	0.21	1.66	0.67	2.86	1.22	No	No	No
Ccl17	C-C Motif Chemokine Ligand 17	1	0.36	1.84	0.44	2.48	1.30	No	No	No
Tgfb2	Transforming Growth Factor Beta 2	1	0.31	2.02	0.61	2.52	0.74	No	No	No
Mstn	Myostatin	1	0.25	1.66	0.54	6.25	4.12	No	No	No
Hc	Hemolytic complement	1	0.51	0.37	0.10	3.53	1.11	No	No	No
Cx3Cl1	C-X3-C Motif Chemokine Ligand 1	1	0.33	0.67	0.31	1.76	0.57	No	No	No
ccl1	C-C Motif Chemokine Ligand 1	1	0.32	0.76	0.39	1.63	0.42	No	No	No
Cxcl5	C-X-C Motif Chemokine Ligand 5	1	0.30	0.29	0.07	1.32	0.24	No	No	Yes
Spp1	Secreted Phosphoprotein 1	1	0.30	36.42	15.78	6.12	1.75	No	Yes	No
Il1rn	Interleukin 1 Receptor Antagonist	1	0.12	15.31	1.83	2.42	0.35	Yes	Yes	Yes

iWAT										
		**Young**		**Old**		**Old-DR**		**Significance**	**Significance**	**Significance**
**Gene**	**Gene Name**	**Fold change**	**SEM**	**Fold change vs young**	**SEM**	**Fold change vs young**	**SEM**	**Young vs Old**	**Young vs Old-DR**	**Old vs Old-DR**
Il12a	Interleukin 12a	1	0.48	0.10	0.05	0.21	0.08	No	No	No
Mstn	Myostatin	1	0.30	0.02	0.01	0.09	0.04	No	No	No
ccl1	C-C Motif Chemokine Ligand 1	1	0.29	1.42	0.99	0.56	0.21	No	No	No
Fasl	Fas Ligand	1	0.17	1.03	0.57	0.66	0.23	No	No	No
Cd40Ig	CD40 Ligand	1	0.30	0.53	0.32	0.14	0.06	No	No	No
Cxcl10	C-X-C Motif Chemokine Ligand 10	1	0.54	1.33	0.96	0.17	0.02	No	No	No
Xcl1	X-C Motif Chemokine Ligand 1	1	0.57	0.90	0.42	0.17	0.05	No	No	No
Il16	Interleukin 16	1	0.22	3.40	0.86	1.37	0.44	No	No	No
Ccl17	C-C Motif Chemokine Ligand 17	1	0.52	4.09	1.07	1.47	0.61	Yes	No	No
Cx3Cl1	C-X3-C Motif Chemokine Ligand 1	1	0.33	7.75	2.92	0.56	0.14	No	No	No
Il7	Interleukin 7	1	0.25	5.23	1.58	0.67	0.19	No	No	No
Hc	Hemolytic complement	1	0.29	4.50	1.59	0.21	0.03	No	No	No
Ppbp	Pro-Platelet Basic Protein	1	0.26	2.19	0.32	1.10	0.44	Yes	No	No
Cxcl12	C-X-C Motif Chemokine Ligand 12	1	0.15	3.59	1.35	0.63	0.11	No	No	No
Il18	Interleukin 18	1	0.19	3.00	0.54	0.60	0.14	Yes	No	Yes
Il15	Interleukin 15	1	0.27	1.89	0.34	0.59	0.06	No	No	Yes
Ccl2	C-C Motif Chemokine Ligand 2	1	0.26	1.62	0.46	0.64	0.14	No	No	No
Il4	Interleukin 4	1	0.17	1.46	0.58	0.61	0.11	No	No	No
Cxcl13	C-X-C Motif Chemokine Ligand 13	1	0.28	3.37	1.06	0.70	0.13	No	No	No
Il27	Interleukin 27	1	0.55	1.77	0.79	0.79	0.20	No	No	No
Il6	Interleukin 6	1	0.39	1.34	0.58	0.82	0.30	No	No	No

Cxcl16	C-X-C Motif Chemokine Ligand 16	1	0.76	1.85	0.49	0.28	0.05	No	No	No
Ccl7	C-C Motif Chemokine Ligand 7	1	0.27	1.12	0.43	0.36	0.16	No	No	No
Ccl3	C-C Motif Chemokine Ligand 3	1	0.29	1.13	0.22	0.30	0.08	No	No	No
Ccl5	C-C Motif Chemokine Ligand 5	1	0.20	0.91	0.55	0.61	0.29	No	No	No
Tnfsf13b	TNF Superfamily Member 13b	1	0.40	1.53	0.59	0.39	0.17	No	No	No
IL10	Interleukin 10	1	0.29	1.12	0.23	0.52	0.09	No	No	No
Cntf	Ciliary Neurotrophic Factor	1	0.30	0.95	0.10	0.57	0.14	No	No	No
Ccl12	C-C Motif Chemokine Ligand 12	1	0.44	1.36	0.57	0.42	0.04	No	No	No
Ccl4	C-C Motif Chemokine Ligand4	1	0.49	0.87	0.22	0.39	0.12	No	No	No
Tnf	Tumor Necrosis Factor	1	0.32	2.54	1.13	1.08	0.24	No	No	No
Ccl19	C-C Motif Chemokine Ligand 19	1	0.16	1.29	0.29	1.13	0.30	No	No	No
Ccl11	C-C Motif Chemokine Ligand 11	1	0.08	1.44	0.29	0.75	0.07	No	No	No
Csf1	Colony Stimulating Factor 1	1	0.07	1.59	0.11	0.56	0.13	No	Yes	Yes
Ccl22	C-C Motif Chemokine Ligand 22	1	0.25	1.96	0.44	0.62	0.20	No	No	Yes
Pf4	Platelet Factor 4	1	0.34	2.16	0.69	1.04	0.28	No	No	No
Cxcl11	C-X-C Motif Chemokine Ligand 11	1	0.42	1.10	0.13	1.18	0.34	No	No	No
Ccl24	C-C Motif Chemokine Ligand 24	1	0.23	0.92	0.36	0.81	0.19	No	No	No
Tnfsf11	TNF Superfamily Member 11	1	0.34	0.89	0.33	0.91	0.25	No	No	No
Ltb	Lymphotoxin Beta	1	0.18	1.21	0.40	1.29	0.31	No	No	No
Tnfsf10	TNF Superfamily Member 10	1	0.12	0.84	0.16	1.05	0.20	No	No	No
Cxcl1	C-X-C Motif Chemokine Ligand 1	1	0.16	0.88	0.31	1.26	0.43	No	No	No

Gpi1	Glucose-6-Phosphate Isomerase 1	1	0.07	0.37	0.05	1.00	0.14	Yes	No	Yes
Il17f	Interleukin 17f	1	0.13	0.27	0.05	1.39	0.20	Yes	No	Yes
Mif	Macrophage Migration Inhibitory Factor	1	0.10	0.70	0.24	0.75	0.16	No	No	No
Cxcl9	C-X-C Motif Chemokine Ligand 9	1	0.17	0.50	0.14	0.64	0.10	Yes	No	No
Ctf1	Cardiotrophin 1	1	0.20	0.80	0.24	0.52	0.08	No	No	No
Vgefa	Vascular Endothelial Growth Factor A	1	0.05	0.71	0.15	0.45	0.13	No	No	No
Il11	Interleukin 11	1	0.34	0.79	0.30	0.34	0.08	No	No	No
Spp1	Secreted Phosphoprotein 1	1	0.19	1.17	0.84	0.83	0.12	No	No	No
Thpo	Thrombopoietin	1	0.14	0.36	0.06	0.60	0.09	Yes	Yes	No
Tgfb2	Transforming Growth Factor Beta 2	1	0.38	0.60	0.15	0.47	0.09	No	No	No
