# Supplementary material for: Necroptosis increases with age and is reduced by dietary restriction
Source: Aging Cell. 2018 Apr 25;17(4):e12770. doi: 10.1111/acel.12770 (PMC6052392; doi:10.1111/acel.12770)
Supplement: Supplementary file 2 [file ACEL-17-na-s002.docx]

**Experimental Procedures**

**Animals***.* All experiments were approved by the Institutional Animal Care and Use Committee at the University of Oklahoma Health Science Center. Male C57Bl/6 mice were obtained from the NIA aging colony: 9- and 24-month-old mice fed *ad libitum* and 24-month-old mice fed a caloric restricted diet. The mice were maintained under SPF conditions in a HEPA barrier environment in the animal facility at the University of Oklahoma Health Sciences Center until sacrifice. *Ad libitum* and DR mice were individually housed and were fed irradiated NIH-31 mouse/rat diet from Teklad (Envigo, Madison, WI). DR was started at 14 weeks of age at 10% restriction, increased to 25% restriction at 15 weeks, and to 40% restriction at 16 weeks where it is maintained throughout the life of the animal.

**Western blotting.** The adipose tissue collected during sacrifice was immediately frozen in liquid nitrogen and stored at −80°C until use. Homogenization of tissues and western blotting was performed as previously described (Bhaskaran *et al*., 2017). The following primary antibodies were used: Anti-MLKL (phospho S345) antibody (Abcam, ab196436); Anti-MLKL antibody (Abcam, ab194699); Anti-RIP3 (phospho S232) antibody (Abcam, ab195117), RIPK3 Antibody (Novus Biologicals, NBP1-77299); RIPK1 antibody (Novus Biologicals, NBP1-77077); Phospho-NF-κB p65 (Ser536) antibody (Cell Signaling Technology, 3033) and NF-κB p65 antibody (Cell Signaling Technology, 6956). To validate the Anti-MLKL (phospho S345) antibody, extracted proteins from samples were run on a gel along with samples treated with necrostatin-1s that is shown to reduce necroptosis and MLKL phosphorylation (Caccamo *et al*., 2017). Images were taken using a G:BOX imaging system (Syngene) and quantified using ImageJ software (U.S. National Institutes of Health, Bethesda, MD, USA).

**Quantitative real-time PCR.** Total RNA was extracted using the RNeasy kit (Qiagen, Valencia, CA, USA) from 50 mg of frozen WAT as described before (Bhaskaran *et al*., 2017). First-strand cDNA was synthesized using SuperScript II reverse transcriptase (Life Technologies, Grand Island, NY, USA) and quantitative real-time PCR was performed with ABI Prism using Power SYBR Green PCR Master Mix with the primers (Applied Biosystems, Foster City, CA, USA). The following primers were used for the study: RIPK1 (forward): 5′-GACTGTGTACCCTTACCTCCGA-3′; RIPK1 (reverse): 5’-CACTGCGATCATTCTCGTCCTG-3’; RIPK3 (forward): 5′-GAAGACACGGCACTCCTTGGTA-3′; RIPK3 (reverse): 5′-CTTGAGGCAGTAGTTCTTGGTGG-3′; MLKL (forward) 5′-CTGAGGGAACTGCTGGATAGAG-3′; MLKL (reverse) 5′-CGAGGAAACTGGAGCTGCTGAT-3′; VPS4A (forward): 5’-GATCTGGTGACAAAAGCCACA-3’; VPS4A (reverse): 5’-CTTTGCTCGAATGCTCTCCTT-3’; VPS4B (forward): 5’-TGGGAACTATGAGGAAGCTCTT-3’; VPS4B (reverse): 5’- ACTTTGCTTGGCTTTATCACCTT-3’; VPS37B (forward): 5’-ACATGGTGCGGGGTATGGA-3’; VPS37B (reverse): 5’-TTTGGGTCAGGCGAGCTTTC-3’, CHMP2A (forward): 5’-AGACGCCAGAGGAACTACTTC-3’; CHMP2A (reverse): 5’- ACCAGGTCTTTTGCCATGATTC-3’; CHMP4B (forward): 5’- GGAGAAGAGTTCGACGAGGAT-3’; CHMP4B (reverse): 5’- TGGTAGAGGGACTGTTTCGGG-3’. Calculations were performed by a comparative method (2^−ΔΔCt^) using β-microglobulin, actin, and 18S. Normalized mRNA level for adult mice is taken as one and the values for old and old-DR mice are represented as fold change.

Gene expression of cytokines and chemokines were was measured using RT² Profiler™ PCR Array Mouse Cytokines & Chemokines (PAMM-150Z) (Qiagen) according to the manufacturer’s instructions. Gene expression was quantified relative to the geometric mean of 5 stable reference genes: beta actin; beta-2 microglobulin; glyceraldehyde-3-phosphate dehydrogenase; beta glucuronidase; and heat shock protein 90 alpha (cytosolic), class B member 1.

**Statistics.** One-way ANOVA with multiple comparisons was used to analyze all data.

**Reference**

Bhaskaran, S., Unnikrishnan, A., Ranjit, R., Qaisar, R., Pharaoh, G., Matyi, S., …. & Deepa, S. S. (2017) A fish oil diet induces mitochondrial uncoupling and mitochondrial unfolded protein response in epididymal white adipose tissue of mice. *Free Radical Biology & Medicine*, *108*, 704-714. https://doi: 10.1016/j

Caccamo, A., Branca, C., Piras, I. S., Ferreira, E., Huentelman, M. J., Liang, W. S., … Oddo, S. (2017). Necroptosis activation in Alzheimer's disease. *Nature Neuroscience*, *20*(9), 1236-1246. https://doi:[10.1038/nn.4608](https://doi.org/10.1038/nn.4608)
